# Girdling promotes tomato fruit enlargement by enhancing fruit sink strength and triggering cytokinin accumulation

**DOI:** 10.3389/fpls.2023.1174403

**Published:** 2023-06-16

**Authors:** Lin Chai, Heng Wang, Hongjun Yu, Endi Pang, Tao Lu, Yang Li, Weijie Jiang, Qiang Li

**Affiliations:** State Key Laboratory of Vegetable Biobreeding, Institute of Vegetables and Flowers, Chinese Academy of Agricultural Sciences, Beijing, China

**Keywords:** carbohydrate allocation, sucrose unloading, sucrose signal, sink strength, trans-zeatin, *Solanum lycopersicum* L.

## Abstract

Girdling is a horticultural technique that enhances fruit size by allocating more carbohydrates to fruits, yet its underlying mechanisms are not fully understood. In this study, girdling was applied to the main stems of tomato plants 14 days after anthesis. Following girdling, there was a significant increase in fruit volume, dry weight, and starch accumulation. Interestingly, although sucrose transport to the fruit increased, the fruit’s sucrose concentration decreased. Girdling also led to an increase in the activities of enzymes involved in sucrose hydrolysis and AGPase, and to an upregulation in the expression of key genes related to sugar transport and utilization. Moreover, the assay of carboxyfluorescein (CF) signal in detached fruit indicated that girdled fruits exhibited a greater ability to take up carbohydrates. These results indicate that girdling improves sucrose unloading and sugar utilization in fruit, thereby enhancing fruit sink strength. In addition, girdling induced cytokinin (CK) accumulation, promoted cell division in the fruit, and upregulated the expression of genes related to CK synthesis and activation. Furthermore, the results of a sucrose injection experiment suggested that increased sucrose import induced CK accumulation in the fruit. This study sheds light on the mechanisms by which girdling promotes fruit enlargement and provides novel insights into the interaction between sugar import and CK accumulation.

## 1 Introduction

Girdling is a common horticultural practice used in trees and some herbaceous crops. It involves the removal of a ring of bark that includes the phloem tissue, which obstructs the transport of assimilates in the phloem but does not directly affect water and mineral transport by the xylem (Van de Wal et al., 2017). Girdling is often applied to adjust the distribution of photosynthates. Usually, following girdling, photoassimilates produced by the leaves are allocated more to the developing flowers or fruits (Tyagi et al., 2020). In fruit trees, girdling has been widely used to regulate plant growth (Castro et al., 2019), increase fruit set (Quentin et al., 2013) and yield (Otero and Rivas, 2017), and improve fruit quality (Singh Brar et al., 2008).

Girdling is also applied in cotton and tobacco production to reduce the abscission of cotton bolls (Dai and Dong, 2011) and improve tobacco quality (Zhao et al., 2018). In tomato plants, girdling is performed after topping to increase fruit size and advance fruit maturity (Chai et al., 2021). This practice has been reported to increase fruit size in several other species, such as grape (Casanova et al., 2009), citrus (Otero and Rivas, 2017), persimmon (Juan et al., 2009), and peach (Agusti et al., 1998). However, the underlying mechanism of how girdling affects fruit size remains obscure.

Tomato (*Solanum lycopersicum* L.) is a significant horticultural crop that is cultivated globally. It has also been a primary model plant for studying fruit development (Azzi et al., 2015). Tomato fruit size is determined by cell number and size, factors which are regulated by cell division and cell expansion processes, respectively (Quinet et al., 2019). These processes are complex and involve intricate interactions between carbon partitioning and hormone signaling (Albacete et al., 2014).

Tomato fruit growth is closely associated with carbohydrate partitioning. Source organs, such as mature leaves, generate carbohydrates through photosynthesis and export fixed carbon in the form of sucrose to sink organs, such as fruits, tubers, and meristems (Osorio et al., 2014). Tomato fruit is an important sink organ that needs to import carbohydrates to meet its metabolic demands and support its growth and development (Albacete et al., 2014). To sustain its growth, the fruit has to compete for carbohydrates. A fruit’s carbohydrate competition capacity is strongly influenced by the fruit sink strength (Smith et al., 2018), which is directly linked to its capacity to unload sucrose from phloem and utilize imported carbohydrates (Osorio et al., 2014). There are two pathways for sucrose unloading: the symplastic pathway and the apoplastic pathway (Patrick, 1997). During tomato fruit development, the sucrose unloading pathway shifts from a symplastic to an apoplastic pathway (Ruan and Patrick, 1995). In the symplastic pathway, sucrose moves along the concentration gradient from sieve element-companion cell (SE-CC) complexes directly to surrounding parenchyma cells (PCs) via plasmodesmata (Patrick, 1997). In the apoplastic pathway, sucrose transported over long distances in the phloem arrives at the fruit and is released into the apoplastic space between cells via sugars will eventually be exported transporters (SWEETS) from phloem sieves or sieve element-companion cell (SE-CC) complexes (Hu et al., 2021). Sucrose can then be directly transported into parenchyma cells (PCs) by sucrose transporters (SUTs), or converted into fructose and glucose by cell wall invertase (CWInv) and then transported into PCs by hexose transporters (HTs) (Slewinski, 2011). In PCs, sucrose can be hydrolyzed by invertase into glucose and fructose, or by sucrose synthase into UDP-glucose and fructose, which can then be metabolized or synthesized into starch (Wang et al., 1993).

Sucrose unloading and hydrolysis play essential roles in determining sink strength, increasing the sucrose concentration gradient from source to sink, and enhancing the sugar utilization of fruits (Braun, 2022). Sugar transporters play a critical role in phloem unloading in fruit, which contributes to fruit sugar accumulation, yield, and quality (Wen et al., 2022). In tomato plants, the expression of the CWInv gene (*LIN5*) was upregulated by the heterologous expression of the apple hexose transporter *MdHT2.2*, leading to significant increases in fructose and glucose levels in mature fruits (Wang et al., 2019). Knockdown of the tomato hexose transporter genes *LeHT1*, *LeHT2*, and *LeHT3* resulted in a 55% decrease in fruit hexose accumulation (McCurdy et al., 2010). *SlSWEET15* is highly expressed in developing fruits and the clustered regularly interspaced short palindromic repeats and CRISPR-associated protein 9 (CRISPR/Cas9)-mediated gene editing of *SlSWEET15* reduced sucrose unloading in fruit, and consequently decreased the size and weight of tomato fruits (Ko et al., 2021). CWInv in tomato plants was found to upregulate the expression of *SlSWEET12c* and *SlHT2*, promoting sucrose efflux and hexose uptake, thereby enhancing fruit development and sugar accumulation (Ru et al., 2020).

*SlSWEET7a* and *SlSWEET14*, which are primarily expressed in peduncles, vascular bundles, and seeds, were found to regulate fruit size and sugar content, with their suppression resulting in increased sugar content and fruit size (Zhang et al., 2021). Heterologous expression of *MdSWEET12a*, mainly expressed in SE-CCs in apple fruits, increased sugar unloading and sugar content in fruit by upregulating the genes related to sugar metabolism and transport (Zhang et al., 2021).

CWInvs play a crucial role in determining sucrose unloading and sink strength in sink organs by hydrolyzing sucrose in the apoplast. Knockdown of the cell wall invertase inhibitor, *SlINVINH1*, has been shown to increase tomato fruit sugar content without reducing fruit weight (Jin et al., 2009; Palmer et al., 2015; Ru et al., 2017; Kawaguchi et al., 2021). Higher CWInv activity in flowers and young fruits partitions more sucrose to fruits and less to vegetative tissues, thereby improving heat tolerance in tomato fruit (Li et al., 2012). Silencing the *LIN5* gene, which encodes a CWInv, and using RNA interference in tomato plants results in an increase in the fruit abortion rate and a reduction in fruit size (Zanor et al., 2009).

Hormone signaling plays an important role in the development of fruit (Albacete et al., 2014). Auxin and gibberellic acid (GA) are the primary regulators of fruit set initiation and growth (Fenn and Giovannoni, 2021). The biosynthesis of auxin in the developing seeds and GAs in the pericarp actively promotes fruit growth by facilitating pericarp cell division and elongation (Quinet et al., 2019). The application of exogenous auxin and GA has been found to induce fruit initiation and stimulate parthenocarpic development (de Jong et al., 2009). Moreover, specific genetic manipulations have provided further insights into the roles of auxin and GA in fruit development. For instance, the silencing of the PIN-FORMED (PIN) auxin efflux transport proteins gene *PIN4* resulted in parthenocarpic fruits being developed due to precocious fruit development (Mounet et al., 2012). In addition, the heterologous overexpression of the citrus *CgGA20ox1* gene in tomatoes led to an elevated GA4 content and the subsequent development of parthenocarpic fruits (Garcia-Hurtado et al., 2012).

Cytokinin (CK) is known to play a significant role in cell division during fruit development. The exogenous application of a synthetic CK, N-(2-chloro-pyridin-4-yl)-N”-phenylurea (CPPU), can induce parthenocarpic tomato fruit development by promoting cell division (Matsuo et al., 2012). Furthermore, it has been observed that endogenous levels of CK also play a role in regulating fruit growth (Mariotti et al., 2011). For instance, in *Arabidopsis*, the downregulation of *CK dehydrogenase* (*CKX*) gene expression resulted in increased CK levels and larger dry fruit size (Hussain et al., 2020). In kiwifruit, the analysis of phytohormones and transcriptomics revealed that endogenous CK affects fruit growth under conditions of carbon starvation (Nardozza et al., 2020). These findings collectively suggest that CK plays a crucial role in fruit growth and expansion. However, the regulation activity and underlying mechanisms of CK in this process have not yet been fully elucidated.

Our previous research revealed that girdling that is carried out after topping can increase tomato fruit size. This increase in fruit size was found to be dependent on the developmental stage of the fruits at the time of girdling. The most significant increases in fruit size were when girdling was conducted during the onset of the enlargement stage (Chai et al., 2021). Building upon these findings, the objective of this study was to investigate the underlying mechanism through which girdling influences fruit size. To minimize interference from other sink organs, we topped tomato plants and retained only one truss with three fruits. Girdling was carried out 14 days after anthesis, and subsequent fruit growth was observed. We conducted an analysis of sucrose unloading, sugar metabolism capacity, and phytohormone content in the tomato fruit. Our sucrose injection experiment also indicated that an increase in sucrose import to the fruits leads to the accumulation of CKs. These findings suggest an interaction between sugar and CK in the regulation of fruit development. Overall, this study provides valuable insights into the mechanisms underlying the promotion of fruit enlargement through girdling, thereby offering meaningful prospects for yield improvement.

## 2 Materials and methods

### 2.1 Plant material and growth conditions

Tomato plants (*Solanum lycopersicum* L.), the cultivar Moneymaker, were used in the experiments. The seeds were germinated in trays filled with a mixture of peat and vermiculite in a 1:1 ratio [volume per volume (v/v)]. When the first four fully expanded leaves appeared, the seedlings were transplanted into individual plastic pots containing 7L of coco coir. These pots were then watered with Hoagland’s solution. The seedlings were grown in a greenhouse located in Beijing, China (40.11°N, 116.16°E) from April to June. The growth conditions during this period were as follows: an average temperature of 25°C during the day and 18°C at night, with 12 to 14 h of light per day. The average light intensity was approximately 300 μmol m^-2^ s^-1^. For the experiment, only the first truss and three flowers that had a similar anthesis time were used. The plants were topped 7 days after the anthesis of the first truss.

### 2.2 Girdling treatment

Girdling was conducted 14 days after anthesis, using a circular cut with a width of 10 mm to remove the phloem tissues, following the procedure described in a previous study (Chai et al., 2021). Plants were girdled on the main stem at 50–100 mm below the first truss, and the girdles were bandaged with grafting membranes. Five leaves were left above the girdle. Non-girdled plants were used as the control group.

### 2.3 Fruit growth measurement and sampling

Immature green fruits were harvested at 0, 3, 6, 9, and 12 days after treatment (DAT). All fruits were harvested between 9 and 10 a.m. with three biological replicates. Each replicate consisted of at least six fruits at each sampling time. After harvesting, the equatorial and vertical diameters of all fruits were promptly measured using digital Vernier calipers, and the fruit volume was calculated in accordance with the method described in Nordey et al. (2015). The fresh weights (FW) of all fruits were also recorded. Subsequently, the fruits were swiftly sliced in half. Half of the fruits were immediately dried (for 1 h at 105°C, and then for 48 h at 55°C) so that the fruit dry weight could be measured. The other half was processed by removing the columellas, placentas, and seeds, and the pericarps were frozen in liquid nitrogen and stored at –80°C for further analysis.

### 2.4 Paraffin section microscopy

At 9 DAT and 12 DAT, the fruits were sliced along the equator. After removing the seeds and pulp, the pericarps were cut at the equatorial level and further divided into approximately 5 mm wide pieces before being immersed in formaldehyde, alcohol, acetic acid (FAA) fixative. These pericarp pieces were then embedded in paraffin and stained with Safranin-O and Fast Green, following a previously described method (Takacs et al., 2012). The slides were observed and photographed using a Leica DFC7000 T microscope (Wetzlar, Germany) equipped with a Nikon DXM 1200 camera. To estimate the pericarp thickness and the number of cell layers, six samples of the pericarp without vascular bundles were examined.

### 2.5 Observation of phloem flow in detached fruits using carboxyfluorescein fluorescence

To analyze fruit sink strength, the phloem flow to detached fruits was observed using 5(6) carboxyfluorescein diacetate (CFDA) across the peduncles of both the girdling treatment and the control group at 3 DAT. CFDA was prepared immediately before use. A stock solution of 1 mM CFDA (dissolved in dimethyl sulfoxide) was diluted with MS solution (2% sucrose) to a working concentration of 20 μM. The peduncles with three fruitlets and a diameter of 2–3 cm each were chosen.

The peduncles were cut near the stem between 9 and 10 a.m. on a sunny day. The cut ends of the peduncles, approximately 0.5 cm in length, were immersed in the CFDA working solution (refer to **Supplementary Figure S1**). We avoided splashing CFDA onto other parts of the peduncles. After a 2-hour incubation period in the darkness, the fluorescence signal of the peduncle cross-sections at the same location (i.e., 1 cm from the first fruit) was observed. More than three peduncles were observed for each treatment. The fluorescence signal was captured using a Leica DFC7000 T fluorescence microscope (Wetzlar, Germany). The images were acquired using a 488 nm excitation laser, maintaining consistent exposure time and gain settings for each image (3.03 ms and 1.4, respectively).

### 2.6 Starch and soluble sugar quantification

Each replicate’s frozen sample was ground into a fine powder using liquid nitrogen. The concentrations of glucose, fructose, and sucrose were determined using UPLC-MS/MS (TQ-S micro, Waters). Approximately 100 mg of the ground frozen fresh pericarp samples were homogenized and extracted in 1,900 μL of water in a water bath at 80°C for 30 min. After sonication (80 W for 10 min) and centrifugation (12,000 rpm for 10 min), the solution was filtered using a 0.22 µm polyethersulfone ultrafiltration membrane as the extraction solution. Subsequently, the extraction solution was mixed with equal acetonitrile for analysis. The UPLC analyses used an ACQUITY UPLC BEH Amide 1.7 μm as the analytical column (2.1 × 100 mm; Waters). In the mobile phase, acetonitrile was utilized as solvent A and 1 mg/mL ammonium hydroxide as solvent B. The temperature of the column and autosampler was 60°C and 10°C, respectively. Initially, the gradient of solvent B ran at 10% and used a flow rate of 0.2 mL/min for 2 min; subsequently, each soluble sugar was separated by linearly increasing the concentration of solvent B from 10% to 20% over 6 min, followed by washing with 10% solvent B for 2 min. The column was re-equilibrated for 5 min in the initial conditions described above. The mass spectrometer was operated in the multiple reaction monitoring (MRM) mode to detect the selective fragments of the [M-H]-precursor ions. Data analysis was carried out using MassLynx ver4.1 (Waters). The total soluble sugar concentration was estimated by summing the concentrations of sucrose, glucose, and fructose.

The remaining pellet obtained after extracting the soluble sugars was utilized to determine the starch concentration. Starch extractions were conducted following the protocol described by Ernesto Bianchetti et al. (2018), with some modifications. Briefly, the supernatants containing the soluble sugar were discarded. The residues were then washed twice with water; after each wash, they were centrifuged for 10 minutes and the supernatant was removed. Subsequently, the remaining pellet was completely dried. The residues were suspended in 3 mL of distilled water, boiled for 15 min, and then acidified with 2 mL of 9.2 mol/L perchloric acid for 15 min. The supernatants were recovered through centrifugation and subjected to the anthrone-sulfuric acid method for measuring the starch concentration. The non-structural carbohydrate concentration was the sum of the total soluble sugar and starch concentrations.

### 2.7 Phloem exudate collection and sucrose concentration relative quantification

Phloem sap was collected from the peduncles of both control and girdling-treated plants at 3 DAT. The peduncles were cut near the stems, and the fruitlets were removed. The experiment was conducted with three biological replicates, and for each replicate, exudate was collected from six peduncles. The ethylenediaminetetraacetic acid (EDTA)-assisted method, as described in the study by Tetyuk et al. (2013), was used to collect phloem exudate. Briefly, the peduncles were immediately placed in tubes containing 20 mM K_2_-EDTA and kept in darkness for 1 h. Afterward, the EDTA solution was discarded, and the peduncles were transferred to sterile water and kept in darkness for 4 h. The phloem sap was frozen in liquid N_2_, lyophilized, and stored at –80°C. For the determination of sucrose concentration, the dried samples of phloem sap were dissolved in 1 mL of sterile water and then analyzed using ultra-performance liquid chromatography-mass spectrometry (UPLC-MS)/mass spectrometry (MS). A relative quantification method was used to calculate the sucrose concentration of the phloem sap. The dried samples were dissolved in sterile water, and their weight after dissolution was used to calculate the final sucrose concentration.

### 2.8 Hormonal analysis

The extraction, purification, and determination of endogenous levels of IAA, GA_3_, trans-zeatin, isopentenyladenine, and dihydrozeatin were performed using the ELISA technique, following the method described in You-Ming et al. (2001). In brief, approximately 500 mg fresh weight (FW) of pericarp was finely ground into a powder using liquid nitrogen, and then subjected to overnight extraction in cold 80% (v/v) methanol at 4°C. The extracts were centrifuged at 4°C. The supernatant was passed through a C_18_ Sep-Pak cartridge (Waters, Milford, MA), eluted, dried in N_2_, and dissolved in phosphate-buffered saline (PBS) for further analysis by ELISA. The mouse monoclonal antigens and antibodies against tZR, iPA, DZR, IAA and GA_3_, and IgG horseradish peroxides used in ELISA were produced at the Phytohormones Research Institute (China Agricultural University). The absorbance was recorded at 490 nm using an ELISA Reader (EL310, Bio-TEX, Winooski, VT). Calculations of the ELISA data were performed as described by Weiler et al. (1981).

As the anti-tZR antibody detects trans-zeatin (tZ) and trans-zeatin riboside (tZR), the CKs quantified by this antibody are described as tZ-type CKs. Similarly, the anti-iPA antibody detects isopentenyl adenosine (iPA) and isopentenyladenine (iP), described as iP-type CKs. The anti-DZ antibody detects dihydrozeatin (DZ) and dihydrozeatin riboside (DZR), described as DZ-type CKs.

### 2.9 Enzyme activity determination

For each enzyme activity assay, approximately 100 mg of FW pericarp samples were finely ground into a powder using liquid nitrogen. The enzyme activities of vacuolar invertase (VacInv), CWInv, cytoplasmic invertase (CytInv), and SUS in the hydrolysis direction were determined using test kits provided by Cominbio Company Ltd. (SAI-2-Y, BAI-2-Y, NI-2-Y, SS-I-2-Y, respectively; Suzhou, China; http://www.cominbio.com/). All procedures were performed in accordance with the instructions provided with the kits. Enzyme activities were measured as the amount of reducing sugars (ug) produced per gram of fresh sample per minute. The total sucrose hydrolysis activity was estimated by summing the enzyme activities of VacInv, CytInv, and SUS.

The activity analysis of ADP-glucose pyrophosphorylase (AGPase) was performed using the AGPase Activity Kit (Cominbio Company Ltd., AGP-2A-Y). The activity of the AGPase enzyme was expressed as μmol NADPH g^-1^ FW min^-1^. The AGPase activity assay was based on the method described by Song et al. (2020). Briefly, together with phosphoglucomutase and glucose-6-phosphate dehydrogenase, AGPase catalyzes the conversion of ADP-glucose into nicotinamide adenine dinucleotide phosphate (NADPH). The activity of AGPase is indicated by the amount of degraded ADP-glucose, which can be quantified by measuring NADPH concentration spectrophotometrically at 340 nm.

### 2.10 Total RNA extraction, complementary DNA synthesis, and quantitative reverse transcription PCR

Total RNA was extracted from pericarp samples using the Trizol reagent (Mei5bio Company Ltd., Beijing, China) following the manufacturer’s instructions. Subsequently, cDNA was synthesized by reverse transcription of 1 μg of total RNA using a cDNA synthesis kit (Vazyme Biotech Company Ltd., Nanjing, China). The RNA and complementary DNA (cDNA) concentrations were determined based on the A_260_ and A_280_ values using a NanoDrop ND-2000 photo spectrometer (Thermo Fisher Scientific, Inc., Waltham, MA, United States). Specific primers for quantitative reverse transcription PCR (RT-PCR) were designed and listed in **Supplementary Table S1**. Quantitative RT-PCR was carried out with a final volume of 15 μL using SYBR qPCR Master Mix (Vazyme Biotech Company Ltd., Nanjing, China) and an iQ5 Multicolor Real-time PCR Detection System (Bio-Rad Laboratories Inc., United States) (Zhang et al., 2016). Melting curves were examined to detect unspecific amplifications and primer dimerization. For relative quantification, *SlACTIN* (Solyc03g078400) was used as an internal reference, and the 2^-△△CT^ method (Livak and Schmittgen, 2001) was used. The threshold cycle (*C_t_*) value was normalized against *SlACTIN* and compared with the control samples. For gene expression analyses, relative expression values were log2-transformed.

### 2.11 Sucrose injection experiments

To investigate the impact of sucrose on the accumulation of cytokinins (CKs), a sucrose injection experiment was conducted. Uniformly sized 15-day-old fruits that were still attached to the plants were injected with sucrose (300 μL, 50 mM) using a sterile 1-mL hypodermic syringe. Mannitol (50 mM) was used as a control. The injection procedure followed the method described by Jia et al. (2013). In brief, the syringe needle was carefully inserted into the fruit core through the peduncle, and the sugar solution was slowly injected into the fruits. Fruits were sampled between 9 and 10 a.m., at 1 and 3 DAT, with three biological replicates. Each replicate consisted of three fruits at each sampling time. The pericarp tissues were rapidly frozen in liquid nitrogen and stored at –80°C until further use.

### 2.12 Statistical analysis

Statistical analysis was conducted using SPSS22.0 software (SPSS Inc., Chicago, IL, USA). Statistical significance was calculated using Student’s t-test analysis with a significance of *p* < 0.05. The graphs were produced using GraphPad Prism 6 software (San Diego, California, USA), and the heat maps were created using TBtools software (v1.09861; https://www.tbtools.com).

## 3 Results

### 3.1 Girdling accelerates fruit expansion

**Figure 1:**
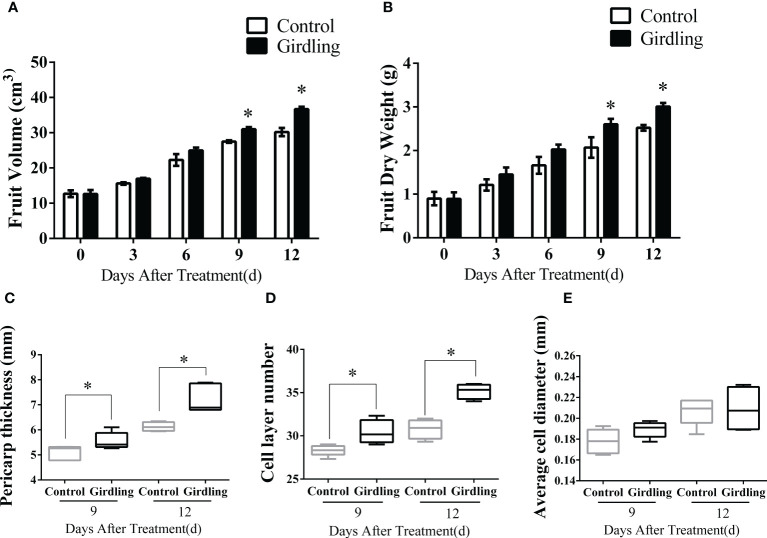
The characteristics of tomato fruit growth. Fruit volume **(A)** and fruit dry weight **(B)** of tomato plants from 0 to 12 DAT. Pericarp thickness **(C)**, the number of cell layers **(D)** and average cell diameter **(E)** of tomato fruit at 9 and 12 DAT. Each value is the mean ± SE of at least three replicates. The asterisks indicate statistical significance according to the results of a Student’s t-test (*p* < 0.05). DAT, days after treatment.

To investigate the impact of girdling on tomato fruit enlargement, the fruit volume and dry weight were measured. Girdling was performed 14 days after anthesis when the fruitlet diameter reached 25 ± 3 mm. As shown in **Figures 1A, B**, both fruit volume and dry weight in the girdling treatment exhibited significant increases compared with the control group. At 12 DAT, the fruit volume in girdling treatment reached approximately 35 cm^3^, surpassing the control group, which measured approximately 30 cm^3^ (**Figure 1A**). Furthermore, following girdling, the fruit dry weight showed a 20% increase compared with the control group at 9 and 12 DAT (**Figure 1B**).

The growth of tomato fruits is influenced by both cell division and cell expansion processes. In this study, we measured the pericarp thickness of fruitlets and the number of cell layers and calculated the average cell diameter at 9 and 12 DAT. Notably, the pericarp thicknesses of girdling fruitlets were significantly greater than those of the control group (**Figure 1C**). Interestingly, this increase in thickness was primarily due to an increase in the number of cell layers rather than an enlargement of individual cell diameter (**Figures 1D, E**). There was an obvious increase in the number of pericarp cell layers in girdling treatment fruits compared with those in the control group at both 9 and 12 DAT. However, there was no significant difference in cell diameter between fruits in the girdling and control groups. Taken together, these results indicated that girdling promoted fruit enlargement and dry mass accumulation, with a more obvious effect on increasing the number of pericarp cell layers.

### 3.2 Girdling increases sucrose import and starch accumulation but decreases sucrose concentration in fruit

**Figure 2:**
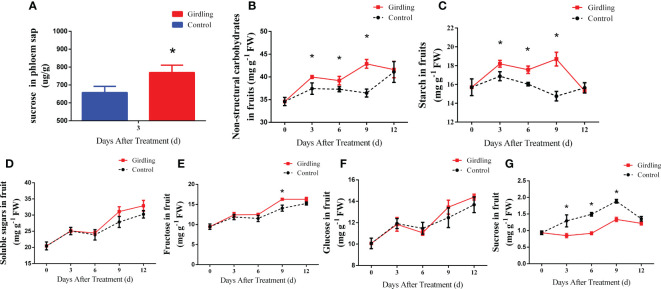
Sugar concentration in tomato fruit. **(A)** Relative quantitation of sucrose concentration in peduncles phloem sap at 3 DAT. **(B–G)** The concentration of non-structural carbohydrate **(B)**, starch **(C)**, total soluble sugar **(D)**, fructose **(E)**, glucose **(F)** and sucrose **(G)** in tomato fruit from 0 to 12 DAT. Each value is the mean ± SE of three replicates. The asterisks indicate a statistical significance according to the results of a Student’s t-test (*p* < 0.05).

The enlargement of tomato fruit is closely associated with sugar import and metabolism, with sucrose being the main form of sugar transported through the phloem. To investigate whether girdling leads to an increased import of sugar into the fruits, the sucrose concentration in the phloem sap of the peduncles was measured at 3 DAT. As shown in **Figure 2A**, the peduncles of fruits in the girdling treatment group exhibited a higher sucrose concentration in the phloem sap compared with the control. This result indicated that after girdling, more sucrose was transported into fruits.

Consistent with the increase of sucrose import, the non-structural carbohydrate concentration in the girdling treatment fruits displayed an obvious increase compared with the control group fruits at 3, 6, and 9 DAT (**Figure 2B**). Similarly, the starch concentration was markedly higher in the girdling treatment fruits compared with the control fruits at the same time points (**Figure 2C**). The total soluble sugar, fructose, and glucose concentrations exhibited a similar increasing trend during fruit growth (14–26 days after anthesis). However, there was no significant difference observed between the girdling and control groups (**Figures 2D–F**). Surprisingly, despite the increased sucrose import to the fruits (**Figure 2A**), the sucrose concentration in the girdling treatment fruits was significantly lower than the control group fruits (**Figure 2G**).

### 3.3 Girdling enhances the enzyme activities of sucrose cleavage and starch synthesis

**Figure 3:**
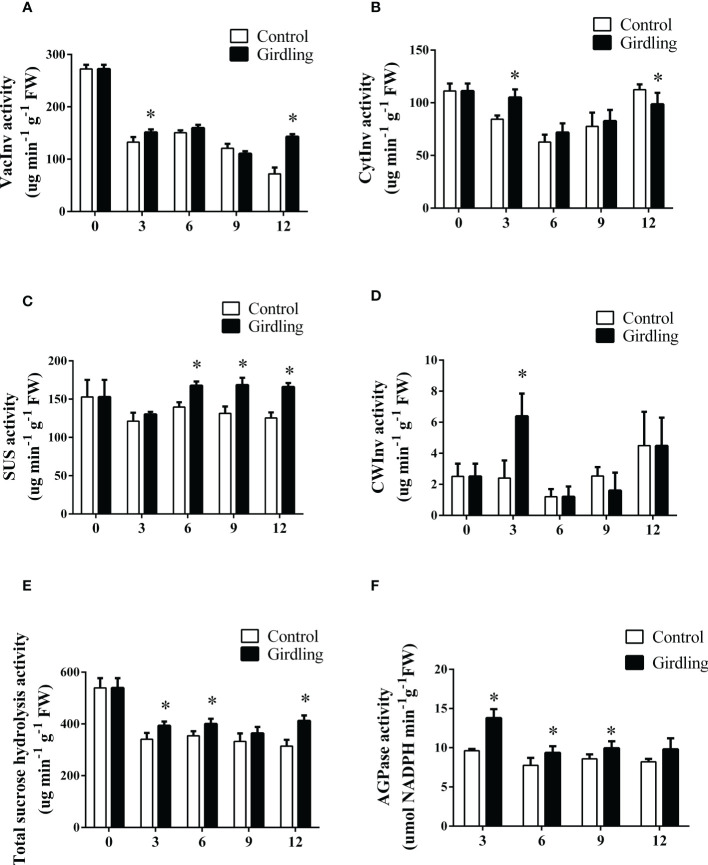
The enzyme activities of sucrose hydrolysis and starch synthesis in tomato fruit. **(A–D)** Enzyme activity of VacInv **(A)**, CytInv **(B)**, SUS **(C)** and CWInv **(D)** in tomato fruit from 0 to 12 DAT. VacInv, vacuolar invertase; CytInv, cytoplasmic invertase; SUS, sucrose synthase; CWInv, cell wall invertase. **(E)** Total sucrose hydrolysis activity in tomato fruit from 0 to 12 DAT. **(F)** AGPase activity in tomato fruit from 0 to 12 DAT. AGPase, ADP-glucose pyrophosphorylase. Each value is the mean ± SE of three replicates. The asterisks indicate statistical significance according to the results of a Student’s t-test (*p* < 0.05).

We hypothesized that the decrease in sucrose concentration in the fruit could be attributed to an enhancement in sucrose hydrolysis. To investigate this hypothesis, we measured the activities of invertase and sucrose synthase (SUS) enzymes in developing fruits. Invertases play a role in sucrose cleavage, converting sucrose into glucose and fructose in both the apoplast (CWInv) and intracellular region (VacInv, CytInv). At 3 DAT, all three invertases exhibited increased activity in the girdling fruits compared to the control (**Figures 3A, B, D**). Particularly, the CWInv activity in girdling fruits was more than twice that of the control at 3 DAT (**Figure 3D**). However, there were no significant differences in invertase activities between the girdling treatment and control groups at 6 and 9 DAT. The enzyme activity of sucrose synthase (SUS), responsible for sucrose cleavage in the cytoplasm, was improved by girdling at 6, 9, and 12 DAT (**Figure 3C**). Furthermore, we calculated the total intracellular sucrose hydrolysis activity, which includes VacInv, CytInv, and SUS. As shown in **Figure 3E**, girdling increased the total intracellular sucrose hydrolysis activity in the fruits from 3 to 12 DAT. These results indicated that girdling promoted the decomposition of sucrose in the fruit.

The enhanced cleavage of sucrose in the fruits can increase the gradient of sucrose concentration from source to sink and facilitate more carbohydrates to be imported into the fruits, thereby promoting cell growth and sugar accumulation (Ji et al., 2020). After girdling, tomato fruits accumulated more starch than the control fruits (**Figure 2C**). Consistent with this, the activity of AGPase, a key enzyme involved in starch biosynthesis, increased noticeably in girdling fruits compared to the control at 3, 6, and 9 DAT (**Figure 3F**). These results suggested that girdling promoted starch synthesis and accumulation during fruit development.

### 3.4 Girdling induces the upregulation of genes involved in sugar transport and utilization in tomato fruits

**Figure 4:**
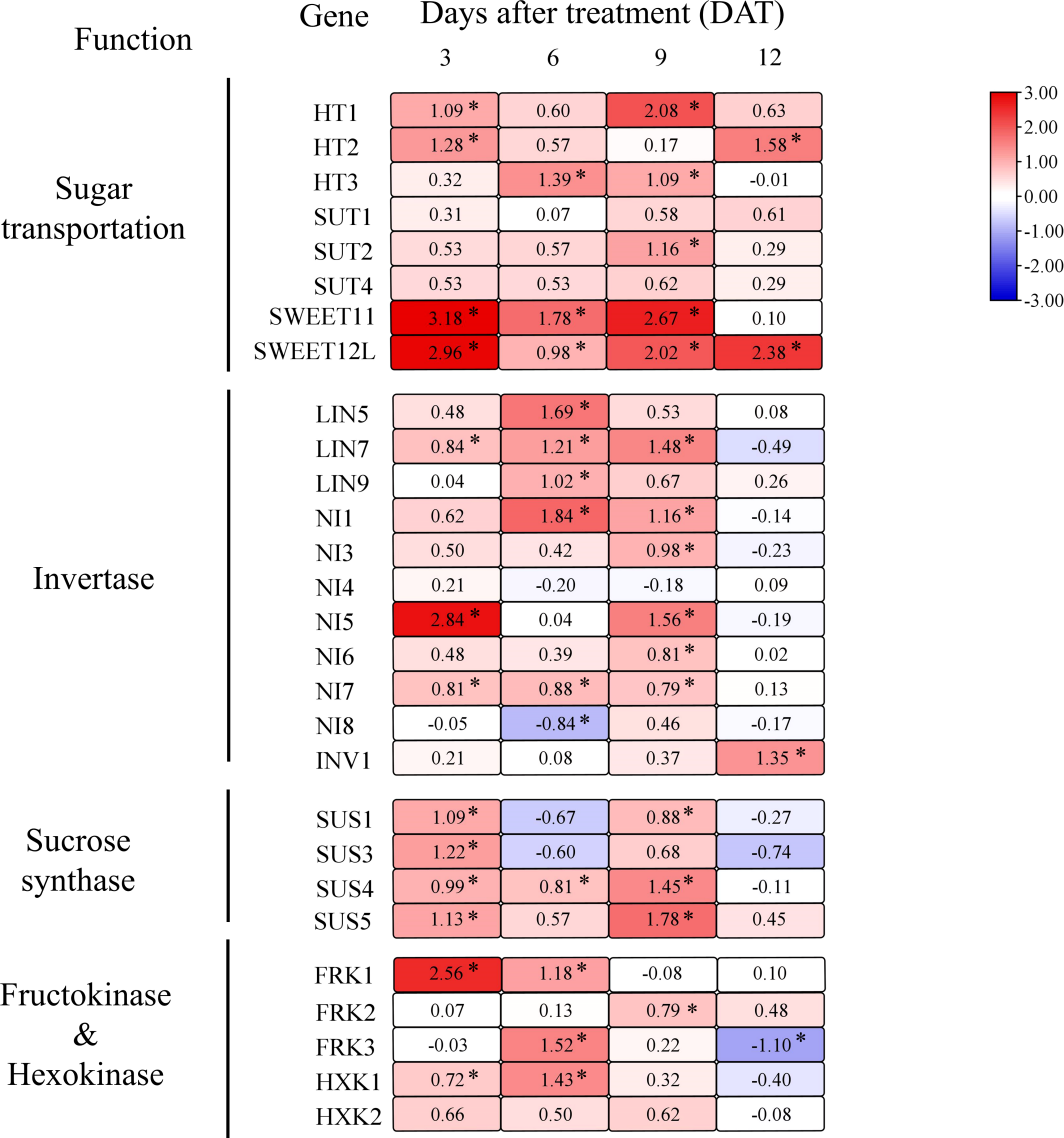
Heat map for genes relative expression levels of sugar transport and utilization in tomato fruit. Effect of girdling on gene relative expression levels involved in sugar transport, invertase, sucrose synthase, fructokinase, and hexokinase in tomato fruit from 3 to 12 DAT. The different colors on the map represent the expression levels of each gene in the girdling treatment relative to that in the control. The value shown in each square is the mean value (*n* = 3) and represents Log2 fold change in gene expression. The asterisks indicate statistical significance according to the results of a Student’s t-test (*p* < 0.05).

To further explore the effect of girdling on fruit sugar metabolism, we measured the transcript levels of key genes involved in sucrose transport and metabolism. Girdling treatment globally increased the expression of genes involved in sugar transport (**Figure 4**). Specifically, the transcript levels of *SWEET11* and *SWEET12L*, which encode tomato SWEET proteins, were elevated in response to girdling. Furthermore, the expression of hexose transporters *HT1/2/3*, responsible for transporting hexose into the cytosol, exhibited increased expression in the tomato fruits under girdling treatment. In addition, the expression of the CWInv genes, *LIN7*, was significantly increased by the girdling treatment.

The expression levels of genes encoding cytoplasmic invertase (*NIs)*, vacuolar invertase (*INV1)*, and sucrose synthase (*SUSs)*, which are responsible for sucrose hydrolysis within the cell, were evidently upregulated following girdling treatment (**Figure 4**). From 3 to 9 DAT, girdling significantly increased the gene expression of cytoplasmic invertase *NI1/5/7* and SUS *SUS1/4/5.* In addition, the expression of vacuolar invertase *INV1* in fruits was enhanced by girdling at 12 DAT.

Sucrose is hydrolyzed by SUS into UDP-glucose and fructose, or by invertase into glucose and fructose. However, neither the fructose nor glucose concentrations showed a significant increase after girdling (**Figures 2B, C**). The lack of increase in hexose concentration may be attributed to the enhanced further metabolic processes involving fructose and glucose. Specifically, glucose and fructose need to be phosphorylated by hexokinase (HXK) or fructokinase (FRK) before undergoing further metabolism (Stein et al., 2016). Therefore, we examined the transcript levels of *FRKs* and *HXKs* and found that their expression was increased following girdling (**Figure 4**). After girdling, the expression of *FRK1* increased noticeably at 3 and 6 DAT, that of *FRK2* increased at 9 DAT, and that of *FRK3* was upregulated at 6 DAT. Moreover, the expression level of *HXK1* was higher at 3 and 6 DAT in girdled fruits than in control fruits. These results suggested that girdling promoted sucrose unloading, sucrose hydrolysis, and subsequent metabolism.

### 3.5 Girdling enhances fruit sink strength

**Figure 5:**
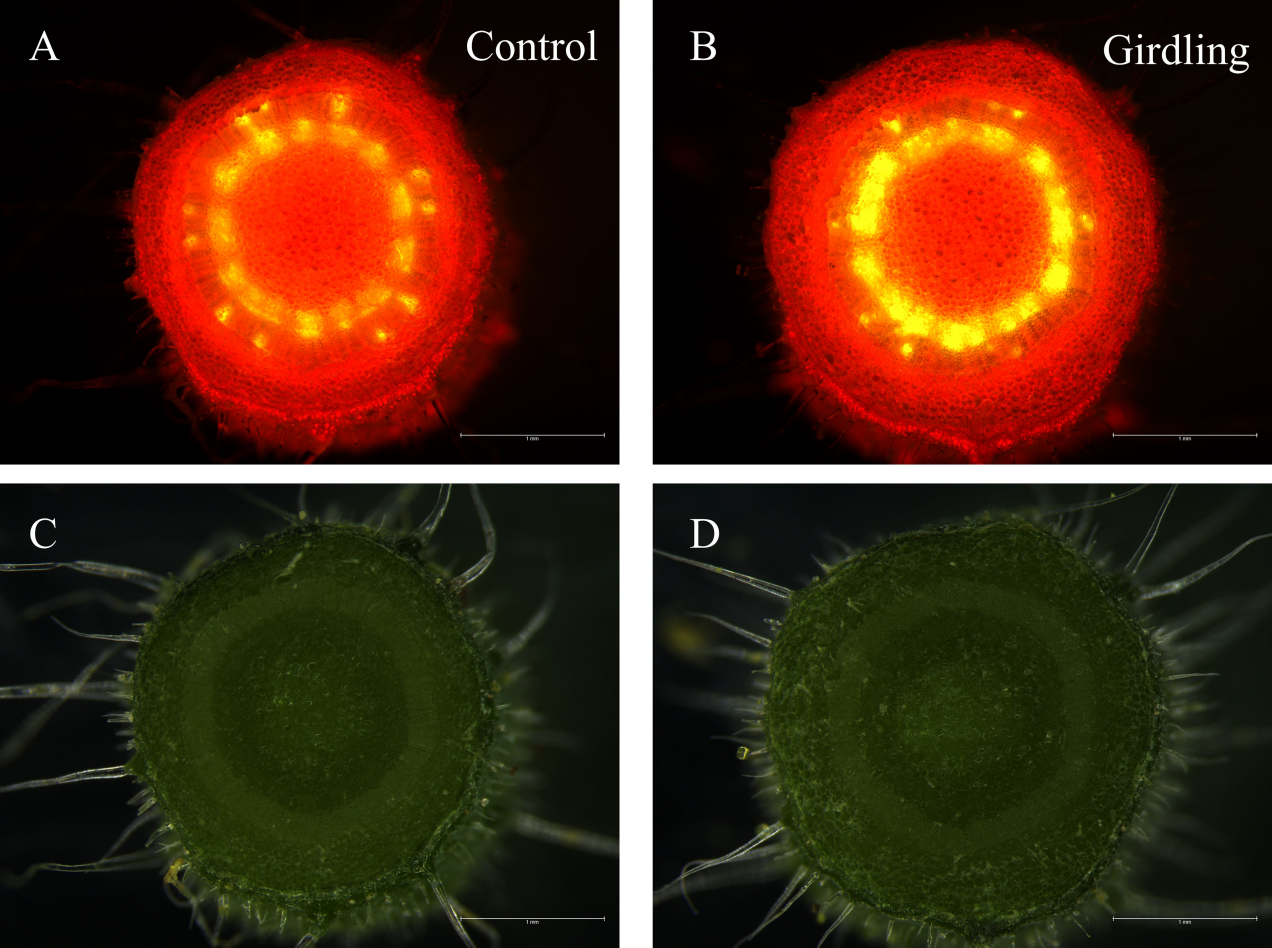
Observation of phloem streaming to tomato fruit using CFDA. CFDA solution (2% sucrose) was applied on the cutting end of the peduncle and the fluorescence signal was observed at the peduncle near the first fruit. Fluorescent image of a free hand cross-section of peduncle in control **(A)** and in girdling treatment **(B)**. Reflected light picture of the same region of control **(C)** and of girdling treatment **(D)**. The fluorescent image was acquired using a 488 nm excitation laser, the exposure time 3.03 ms, and gain number 1.4.

Fruit sink strength is directly linked to the fruit’s capacity to unload sucrose and utilize imported carbohydrates (Osorio et al., 2014). Considering the results that girdling improved enzyme activities and expression of genes related to sucrose unloading and sugar metabolism, we investigated whether girdling enhanced fruit sink strength. Sink strength can also be reflected in the competition for carbohydrates. Therefore, we designed an experiment to observe phloem flow in detached fruits. Carboxyfluorescein (CF) was used as a tracer and applied to the cutting end of peduncles, and the fluorescence signal was observed near the first fruit (**Supplementary Figure S1**). As shown in **Figure 5** and **Supplementary Figure S2**, 2 h after CFDA application, both girdling and control peduncles exhibited bright green fluorescence. Based on the observation and quantification of fluorescence intensity, the peduncle of the girdling treatment exhibited a stronger fluorescence signal compared with the control (**Figure 5** and **Supplementary Figure S3**). This suggests that the fruits under girdling treatment have a greater ability to compete for carbohydrates. Overall, these findings indicate that girdling treatment enhances the sink strength of the fruits.

### 3.6 Girdling triggers CK accumulation in fruits

**Figure 6:**
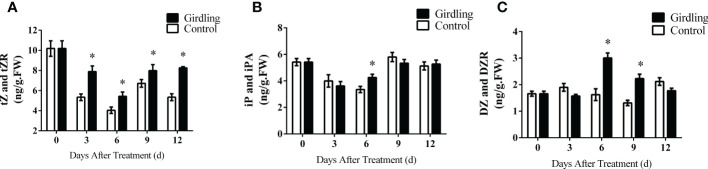
Endogenous levels of CK in tomato fruit. The concentrations of tZ and tZR **(A)**, iP and iPA **(B)**, and DZ and DZR **(C)** in tomato fruit from 3 to 12 DAT. tZ and tZR, trans-zeatin type cytokinin; iP and iPA, isopentenyladenine type cytokinin; DZ and DZR, dihydrozeatin type cytokinin. Each value is the mean ± SE of three replicates. The asterisks indicate statistical significance according to the results of a Student’s t-test (*p* < 0.05). CK, cytokinin.

Phytohormones play a crucial role in regulating fruit set and development. We measured the concentrations of auxin (IAA), gibberellin (GA_3_), and CKs, which are important for tomato fruit development. While there were some differences in the concentrations of auxin and gibberellins at certain time points, they did not exhibit a clear trend of variation (**Supplementary Figure S4**). Therefore, we focused on the changes in CK in this study. The concentrations of CKs and their corresponding ribosides were assessed. Following girdling treatment, there was a significant increase in the levels of tZ and tZR at 3, 6, 9, and 12 DAT (**Figure 6A**). The concentrations of iP and iPA showed limited response to girdling, with iP and iPA levels being higher in girdling treatment fruits than control fruits only at 6 DAT (**Figure 6B**). Girdling fruits exhibited a noticeable increase in DZ and DZR concentrations compared with the control at 6 and 9 DAT (**Figure 6C**). These results demonstrated that girdling treatment promoted the accumulation of CKs, particularly the tZ-type, in the developing fruits.

**Figure 7:**
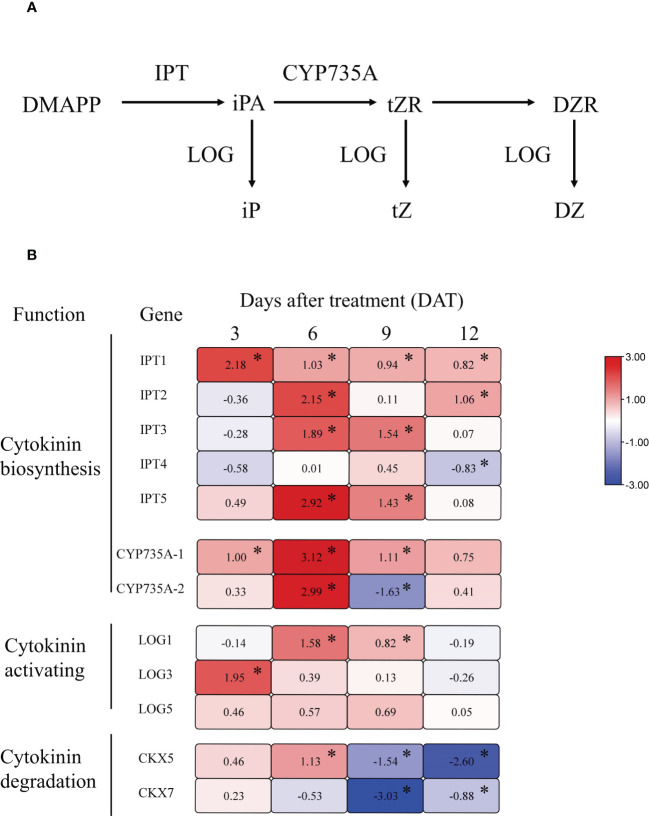
The relative expression levels of cytokinin metabolism-related genes in tomato fruit. **(A)** Schematic representation of the major steps involved in cytokinin biosynthesis. **(B)** Heat map for genes relative expression levels involved in cytokinin synthesis, activation, and degradation in tomato fruit from 3 to 12 DAT. The different colors on the map represent the expression levels of each gene in the girdling treatment relative to that in the control. The value shown in each square is the mean value (*n* = 3) and represents Log2 fold change in gene expression. The asterisks indicate statistical significance according to the results of a Student’s t-test (*p* < 0.05).

To further analyze the effect of girdling on CK metabolism, we measured the expression of key genes involved in CK synthesis, activation, and degradation in girdling-treated and control fruits. Following girdling, we observed a global upregulation of genes encoding adenosine phosphate-isopentenyltransferase (IPT), which catalyzes the initial step of CK biosynthesis (**Figure 7B**). Specifically, the expression of *IPT1* was significantly upregulated at all measured time points, whereas those of *IPT2*, *IPT3*, and *IPT5* were significantly increased only at 6 DAT. *CYP735A1* and *CYP735A2*, encoding cytochrome P450 monooxygenases responsible for converting iP-nucleotides into trans-zeatin (tZ) nucleotides, also exhibited increased transcript levels at 6 DAT (**Figure 7B**). Particularly, the expression of *CYP735A1* was significantly upregulated from 3 to 9 DAT. Moreover, girdling increased the expression of three CK-activating enzyme genes, *LOG1*, *LOG3*, and *LOG5*, from 3 to 9 DAT, although no significant difference was observed in *LOGs* expression at 12 DAT compared with the control (**Figure 7B**). In addition, the CK oxidase/dehydrogenase genes (*CKXs)*, which catalyze the irreversible degradation of CKs, exhibited decreased expression in the girdling treatment group at 9 and 12 DAT, whereas *CKX5* demonstrated increased expression at 6 DAT (**Figure 7B**). Altogether, these results showed that girdling upregulated the transcript levels of genes involved in CK synthesis and activation, and downregulated the genes associated with CK degradation in the fruits.

### 3.7 Exogenous sucrose injection induced CKs accumulation in fruits

**Figure 8:**
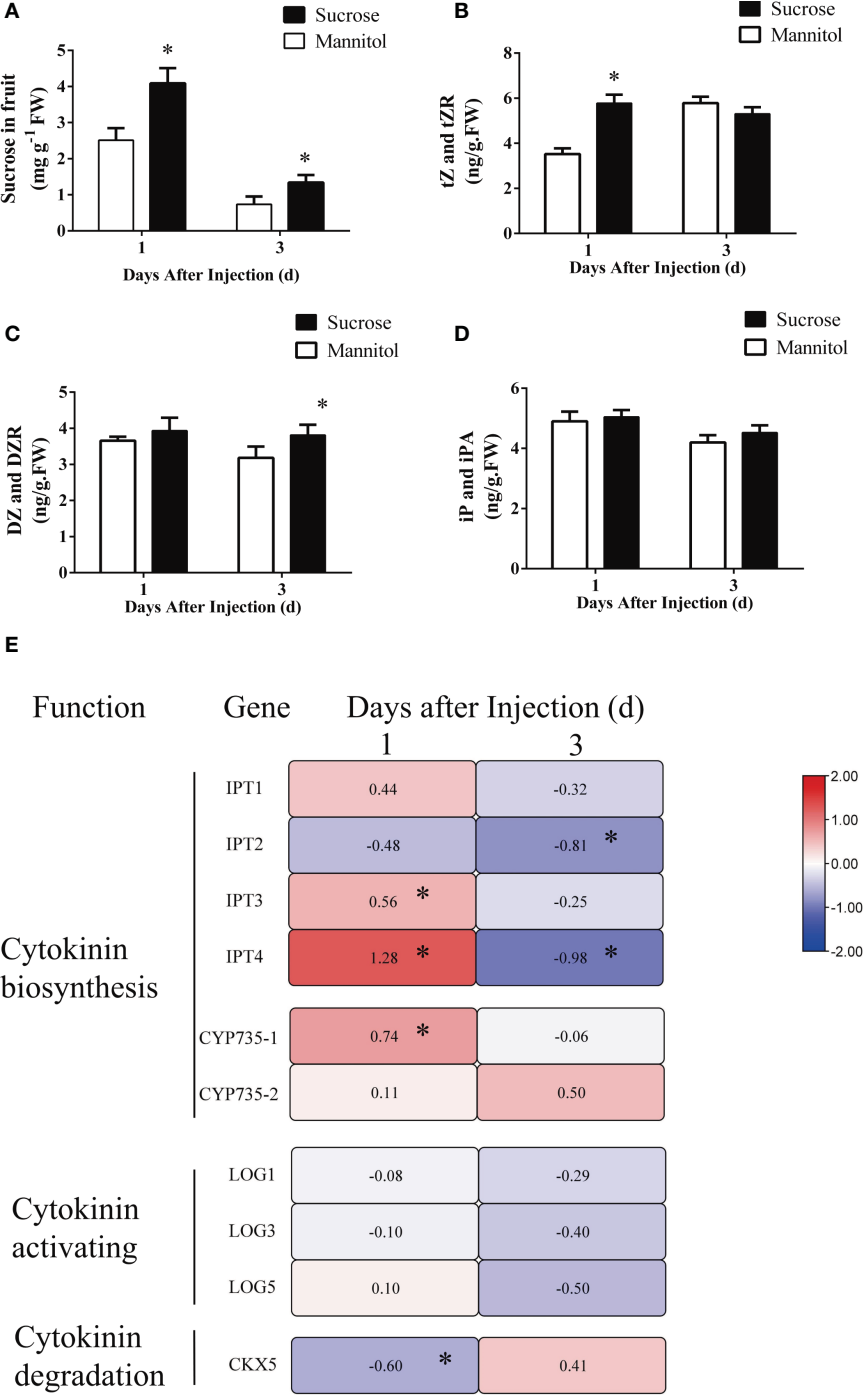
The effect of sucrose injection on the concentration of endogenous cytokinins and the expression of cytokinin metabolism-related genes in tomato fruit. **(A–D)** The concentration of endogenous sucrose **(A)**, tZ and tZR **(B)**, iP and iPA **(C)**, and DZ and DZR **(D)** at 1 and 3 days after mannitol or sucrose injection. tZ and tZR, trans-zeatin type cytokinin; iP and iPA, isopentenyladenine type cytokinin; DZ and DZR, dihydrozeatin type cytokinin. Each value is the mean ± SE of three replicates. The asterisks indicate a statistical significance according to the results of a Student’s t-test (*p*< 0.05). **(E)** Heat map for genes’ relative expression levels involved in cytokinin synthesis, activation, and degradation in tomato fruits at 1 and 3 days after injection. The different colors on the map represent the expression levels of each gene in the sucrose injection relative to that in the mannitol injection. The value shown in each square is the mean value (*n* = 3) and represents Log2 fold change in gene expression. The asterisks indicate a statistical significance according to the results of a Student’s t-test (*p* < 0.05).

Girdling facilitates the phloem transport to fruits, thus facilitating the increased import of sucrose into them. Furthermore, girdling promotes the accumulation of CKs in fruitlets. To determine whether the increased sucrose import resulting from girdling induces CK accumulation in fruits, we injected sucrose directly into the fruits while they remained attached to the plants. This experiment simulated the sucrose import observed after girdling. Mannitol-injected fruits (50 mM) were used as controls. Sucrose concentration significantly increased in sucrose-injected fruits at 1 and 3 DAT compared with mannitol-injected fruits (**Figure 8A**). The concentrations of tZ and tZR in sucrose-injected fruit were noticeably elevated 1 day after injection but showed no difference 3 days after injection Compared with mannitol-injected fruit (**Figure 8B**). The concentrations of DZ and DZR were significantly increased in sucrose-injected fruits 3 days after injection compared with mannitol-injected fruits but had no obvious difference 1 day after injection (**Figure 8C**). At both time points, sucrose injection did not significantly increase iP and iPA concentrations (**Figure 8D**).

To further identify key genes that contribute to CK accumulation in response to sucrose import, the transcript level of key genes involved in CK synthesis and metabolism was detected. At time point 1 day after treatment, injection of sucrose upregulated genes involved in CK synthesis, such as *IPT3, IPT4*, and *CYP735A-1* (**Figure 8E**). No significant difference in the expression levels of *LOGs* was found between sucrose- and mannitol-injected fruits 1 day after injection (**Figure 8E**). In addition, the expression of the CK degradation gene *CKX5* was decreased by sucrose injection (**Figure 8E**). However, at 3 days after injection, *IPT2/4* exhibited declining expression in response to sucrose injection (**Figure 8E**).

The changes in expression levels of *IPT3/4* and *CYP735A-1* were consistent with the changes in tZ and tZR concentrations in sucrose-injected fruits. These findings suggest that injecting sucrose triggered the accumulation of tZ and tZR in fruits, and also transiently upregulated the transcript levels of CK synthesis genes.

## 4 Discussion

### 4.1 Girdling not only increases carbohydrate transport to fruit but increases fruit sink strength

Girdling is a widely used horticultural practice for regulating the distribution of photosynthates. It increases the allocation of carbohydrates produced by leaves to developing flowers and fruits, resulting in improved fruit set (Tyagi et al., 2020), yield (Juan et al., 2009), and quality (Singh Brar et al., 2008). The effect of girdling on increasing fruit size has also been widely reported (Agusti et al., 1998; Singh Brar et al., 2008; Casanova et al., 2009). In our previous study, we found that girdling increased tomato fruit size by altering carbohydrate distribution, with more sugar being partitioned to fruits and less to roots (Chai et al., 2021). However, certain mechanisms still require investigation, such as the metabolic processes induced by girdling, and the response of fruits to the sudden surge in sugar import.

In this study, tomato plants were topped and only one truss with three fruits was left, followed by girdling at 14 days after anthesis. After girdling, fruits exhibited a larger volume and had accumulated more starch and dry mass compared with control fruits (**Figures 1A, B**, **2C**). Moreover, the phloem sap assay of the peduncles revealed that girdling increased the transport of sucrose to the fruits (**Figure 2A**). In tomato plants, sucrose is the primary form of carbohydrate transport, produced in photosynthetically active leaves and translocated to sink tissues via the phloem (Wang et al., 2019). Although sucrose import to the fruits was enhanced, the sucrose concentration of the girdling fruits was significantly lower than that of the control fruits (**Figure 2G**).

Sucrose that moves through the phloem and into sink cells needs to be unloaded, which is crucial for carbohydrate partitioning. Girdling has been shown to increase the expression of genes such as *SWEET11, SWEET12L*, cell-wall invertase gene *LINs*, and hexose transporter *HTs* (**Figure 4**). In addition, there was a noticeable increase in CWInv activity in girdled fruit at 3 DAT (**Figure 3D**). These increased enzyme activity and gene expression that are involved in sugar transport suggested that girdling increased the unloading of sucrose in the apoplastic pathway of tomato fruit. Sucrose unloading plays an important role in fruit development, contributing to fruit sugar accumulation and fruit size. The CRISPR/Cas9-mediated gene editing of *SlSWEET15* reduced sucrose unloading in fruit and consequently decreased the size and weight of tomato fruits (Ko et al., 2021). Overexpression of the sucrose transporter gene *PbSUT2* from pears increased sucrose content but decreased hexose content in tomato fruits (Wang et al., 2016).

We observed that the decrease in sucrose concentration in the fruit was primarily caused by the increase in enzyme activities and gene expression involved in sucrose hydrolysis (**Figures 3**, **4**). This enhanced hydrolysis of sucrose in the sink organs can promote cell growth and sugar accumulation (Ruan et al., 2010). This effect was further supported by the inhibition of cytosolic invertase NI6 through RNA interference (RNAi), which resulted in decreased sugar content and yield in tomato fruits (Coluccio Leskow et al., 2021). Antisense inhibition of *SUS* decreased the sucrose unloading capacity and starch accumulation in tomato fruits (D'Aoust et al., 1999). Overexpression of a potato *SUS* gene in cotton enhanced early seed development and improved yield (Xu et al., 2012). Moreover, decreasing sucrose concentration in the fruit could increase the sucrose concentration gradient from source to sink. This, in turn, may result in more sucrose being transported into the fruit through long-distance phloem transport.

Our results indicated that girdling increased sucrose unloading and hydrolysis in developing tomato fruits. However, there was no significant difference in glucose and fructose concentration between the girdling and control groups (**Figures 2E, F**). The lack of change in hexose concentration, despite the increase in sugar import into fruits, can be potentially explained by enhanced hexose metabolism. As shown in **Figure 4**, the expression levels of *HXK1* and *FRK1* are upregulated in response to girdling treatment. This suggests that the utilization of hexoses within the fruit was enhanced after girdling. Furthermore, girdling evidently increased starch concentration (**Figure 2C**) and elevated APGase activity (**Figure 3F**) in fruits. Glucose and fructose serve as substrates for starch biosynthesis within the fruit. It is possible that the increased hexose is being utilized for starch synthesis. Tomato fruit typically accumulates starch at an early growth stage, and starch can be a means of carbon storage when sugar concentration increases in the sink organs (Osorio et al., 2014). Tomato fruits with higher AGPase activity exhibited higher starch content in the immature fruit, resulting in higher total soluble solids and bigger fruit size at the mature stage (Petreikov et al., 2006). Taking into account the results of carbohydrate concentration, enzyme activities, and gene expression, our findings demonstrated that girdling enhanced sugar utilization and starch synthesis in fruits.

The sink strength of fruits refers to the ability of fruits to take up and store carbohydrates. Our results suggest that girdling enhances the capacities of sugar unloading and utilization in tomato fruit, as demonstrated by the stronger ability of girdled fruits to take up carbohydrates, which was in turn evidenced by the assay of CF fluorescent signal in detached fruits (**Figure 5**). Taken together, girdling increased the sink strength of fruits. Fruit sink strength is not fixed, but can change in response to the environment and source-sink balance (Yu et al., 2015; Ji et al., 2020). The immediate effect of girdling is the increase in sucrose import to fruits. Sucrose is not only a source of carbon and provides energy, but also plays a signaling role (Figueroa and Lunn, 2016). We speculate that the sudden increase of sucrose may act as a signal that actively regulates C storage and sink strength.

### 4.2 Sucrose import induces CK accumulation in fruit

In the present study, we observed that girdling also affected CK content, especially trans-zeatin content, in tomato fruits (**Figure 6**). Besides auxin and gibberellic acid, which are the primary phytohormones in the regulation of fruit growth, CK is also known to influence cell division and expansion (Fenn and Giovannoni, 2021). The application of exogenous CK to unpollinated tomato ovaries induced parthenocarpic fruit development via the promotion of cell division (Matsuo et al., 2012). Similarly, in kiwifruit, CK drives fruit cell expansion and growth, and reduced CK accumulation leads to the downregulation of genes encoding Expansins (Nardozza et al., 2020). Consistent with these findings, our results showed that girdling increased CK accumulation and pericarp cell layer numbers (**Figure 1D**).

In developing fruits, CK has been found to regulate carbohydrate metabolism and transport. Exogenous application of CK increased enzyme activities of CWInv and SUS, and improved sink strength (Albacete et al., 2014). On the other hand, sugar is involved in regulating CK synthesis in developing fruits. In carbon-starved kiwifruit, a six-fold reduction in CK concentration was observed, but other hormones were less affected (Nardozza et al., 2020). In addition, sucrose and CK are known to interact with each other in various plant tissues. In potatoes, sucrose feeding to detached stems promotes the accumulation of CK, and detached stems treated with CK induced vacuolar invertase activity (Salam et al., 2021). In rice, the sucrose transport regulator OsDOF11 protein and CK establish a feedback loop: OsDOF11 directly regulates *OsCKX4* to mediate CK signaling and CK induces the transcript level of *OsDOF11* (Wu et al., 2022).

In our research, both girdling and sucrose injection induced trans-zeatin type CK accumulation. The expression levels of CK biosynthetic enzymes such as isopentenyltransferases (IPTs) and CYP735 were upregulated after girdling and sucrose injection (**Figures 7**, **8**). However, CK accumulation in tomato fruit may not be directly related to sucrose concentration. According to our experimental results, the sucrose concentration in girdled fruits was lower than that in control fruits (**Figure 2G**), whereas the sucrose concentration in sucrose-injected fruit was higher than that in the control (**Figure 8A**). However, the CK concentration in the fruit of both experiments increased. Therefore, we speculate that increased sucrose import, rather than increased sucrose concentration, resulted in CK accumulation. Girdling and sucrose injection both increased the import of sucrose into the fruit, thereby inducing an increase in CK concentration. Sucrose injection is an instantaneous process in which a large amount of sucrose enters the fruit. The effect of increased sucrose import can be detected in the short term, but this effect becomes less significant as time passes after injection. This can explain why, although sucrose concentration was still higher in mannitol-injected fruits than control fruits after 3 days of sucrose injection, there was no difference in the trans-zeatin concentration of both groups (**Figure 8**).

These results suggest that there is a positive feedback loop between sucrose import and CK accumulation in fruit in response to changes in carbohydrate supply. Increasing sucrose import to fruit triggered CK synthesis and accumulation. On the other hand, CK promoted cell division and increased cell layer numbers, which further enhanced sucrose import and fruit sink strength. In this study, we observed that an increase in sucrose import led to CK accumulation; however, the detailed mechanisms underpinning this process still needs to be further explored.

## 5 Conclusions

The present study provided new insights into the mechanisms by which girdling increases fruit size. Girdling was found to enhance sucrose import into the fruit, which in turn led to an improvement in fruit sink strength by increasing the sucrose unloading, hydrolysis, and sugar metabolism capacities of the fruit. Moreover, the increase in sucrose imports triggered CK accumulation, which in turn promoted cell division. These findings indicate the presence of a positive feedback loop between sucrose import and CK accumulation in fruit, in response to changes in carbohydrate supply.

## Data availability statement

The original contributions presented in the study are included in the article/**Supplementary Material**. Further inquiries can be directed to the corresponding authors.

## Author contributions

WJ, HY, and QL contributed to the conception and design of the study; LC, HW, and EP conducted the experiments and collected the data; LC and HW performed the statistical analysis; LC wrote the first draft of the manuscript; HW, TL, and YL contributed to the revision of the manuscript. All authors contributed to the article and approved the submitted version.
