# Supplementary material for: Girdling promotes tomato fruit enlargement by enhancing fruit sink strength and triggering cytokinin accumulation
Source: Front Plant Sci. 2023 Jun 16;14:1174403. doi: 10.3389/fpls.2023.1174403 (PMC10312241; doi:10.3389/fpls.2023.1174403)
Supplement: Supplementary file 2 [file DataSheet_2.docx]

The shareable link of the original files for microscopy images：

https://www.jianguoyun.com/p/DeXICnAQ1Ka8CxjCpfoEIAA
