# Supplementary material for: Girdling promotes tomato fruit enlargement by enhancing fruit sink strength and triggering cytokinin accumulation
Source: Front Plant Sci. 2023 Jun 16;14:1174403. doi: 10.3389/fpls.2023.1174403 (PMC10312241; doi:10.3389/fpls.2023.1174403)
Supplement: Supplementary file 3 [file DataSheet_3.docx]

Supplementary Material

Girdling promotes tomato fruit enlargement by enhancing fruit sink strength and triggering cytokinin accumulation

Lin Chai^1,^ ^†^, Qiang Li^1, †^, Heng Wang^1^, Endi Pang^1^, Tao Lu^1^, Yang Li^1^, Hongjun Yu^1,^ *, Weijie Jiang^1,^ *

^1^ State Key Laboratory of Vegetable Biobreeding, Institute of Vegetables and Flowers, Chinese Academy of Agricultural Sciences, Beijing 100081, China

^†^ These authors share first authorship.

*** Correspondence:**Weijie Jiang

jiangweijie@caas.cn (Weijie Jiang)

Hongjun Yu

yuhongjun@caas.cn (Hongjun Yu)

There are one table and four figures in supplementary material.

# Supplementary Table

**Supplementary Table S1. Gene ID and primers**

| Actin | Solyc03g078400 | Forward Primer | ATCCCAAGGCCAACAGAGAG |
| --- | --- | --- | --- |
|  |  | Reverse Primer | CGACCGCTAGCATACAGAGA |
| HT1 | Solyc02g079220 | Forward Primer | CTGCCATGGGTGGTCTCATT |
|  |  | Reverse Primer | AGGGCAGCCAAATACAACGA |
| HT2 | Solyc09g075820 | Forward Primer | TTCTCCTTGGTTGTGGCGTT |
|  |  | Reverse Primer | GGCTGTTCCGTAGTTGACGA |
| HT3 | Solyc07g006970 | Forward Primer | AGCACCGGCTTTCGTTATGA |
|  |  | Reverse Primer | GATGCTTTACCGAACGTGCC |
| SUT1 | Solyc11g017010 | Forward Primer | TGGATTGCGTGGTTTCCCTT |
|  |  | Reverse Primer | ACAGTTTCGCATCACCGACT |
| SUT2 | Solyc05g007190 | Forward Primer | TTGGCTATGCGGTCCTA |
|  |  | Reverse Primer | GTGCCTTTGAAAGTGCTG |
| SUT4 | Solyc04g076960 | Forward Primer | TGTGGACCGCTTTCAGGTTT |
|  |  | Reverse Primer | GACAATAAACGGACGCCGAC |
| SWEET11 | Solyc03g097870 | Forward Primer | AGTCATTTGCTTGGAATTTGGCA |
|  |  | Reverse Primer | AAGCCATTGTAACGGCCATCT |
| SWEET12L | Solyc03g097590 | Forward Primer | AGTGCTGTTATGTGGTTCTTCT |
|  |  | Reverse Primer | AGTCCCAAATTAAGCCACGG |
| SUS1 | Solyc12g009300 | Forward Primer | GCGTGTGCTAGAGATGGTGT |
|  |  | Reverse Primer | CCAAGGCGGGAACTTGATCT |
| SUS3 | Solyc07g042550 | Forward Primer | TCCTCCATCCATTACTTCCCTC |
|  |  | Reverse Primer | ATCCCTTTCCCGTGGCTTTC |
| SUS4 | Solyc09g098590 | Forward Primer | AGCAGTTGATGAGAAGCAGTGA |
|  |  | Reverse Primer | CAACCGTGCTTGCCTGTTTA |
| SUS5 | Solyc03g098290 | Forward Primer | AAGCCAGAGGAAGCTCAACC |
|  |  | Reverse Primer | GCACTGTCTCCCCATCCTTT |
| NI1 | Solyc01g100810 | Forward Primer | TTTATTCCTTCCGCCCTCGC |
|  |  | Reverse Primer | AGTCAACAGGTGCTACACGG |
| NI3 | Solyc11g067050 | Forward Primer | TGAGGATGAAGCGTGGCATT |
|  |  | Reverse Primer | GTACGCACTTTGAAGCTGGC |
| NI4 | Solyc11g020610 | Forward Primer | GCACGATACAGAAGGGGGAG |
|  |  | Reverse Primer | ACCACCACGTTTAGGCACAA |
| NI5 | Solyc01g111100 | Forward Primer | CGTCGAATGTATTGTTGAGCC |
|  |  | Reverse Primer | AATGCTCCAAAGCACGGAAAA |
| NI6 | Solyc11g007270 | Forward Primer | GTCAAGGTCTGATGCCTGCT |
|  |  | Reverse Primer | CCAGAGGACTTCCCATACGC |
| NI7 | Solyc06g065210 | Forward Primer | GATGCTCTACGCCGCACTAT |
|  |  | Reverse Primer | CAGGCATAACCCCTTCTCCC |
| NI8 | Solyc04g081440 | Forward Primer | AGTTGGAGAAATGCCGCTGA |
|  |  | Reverse Primer | TCAGCTAGTTCAATGGCCCG |
| LIN5 | Solyc09g010080 | Forward Primer | TCCGCGACAAGTATGGCTAA |
|  |  | Reverse Primer | TGTTCGGCCTCGTTCAAACT |
| LIN7 | Solyc09g010090 | Forward Primer | TCAATGGCCTGTGGAGGAAC |
|  |  | Reverse Primer | TGCACCCTTGAGTCCACAAA |
| LIN9 | Solyc08g079080 | Forward Primer | GCCCGTGGCTGAAGTTGATA |
|  |  | Reverse Primer | AATGGTCCTAATGCCCCACG |
| INV1 | Solyc03g083910 | Forward Primer | CGCTACCATCCTACCCGATG |
|  |  | Reverse Primer | GTCCAAGCAGTAGTCGGGTC |
| FRK1 | NM_001246964.2 | Forward Primer | CCAATGTTTGTGCTGCCCTG |
|  |  | Reverse Primer | CCAACAGATGCCAGAGACGA |
| FRK2 | NM_001246959.2 | Forward Primer | CGACGATGAGTTCGGTCACA |
|  |  | Reverse Primer | GCTCTACATGGCTCCACGAT |
| FRK3 | NM_001247467.2 | Forward Primer | TGCAAAGGATGCTGGTGTGA |
|  |  | Reverse Primer | TGGACCTTCTGTGACGAGGA |
| HXK1 | NM_001247028.1 | Forward Primer | TATGCGGTGGACAAAAGGCT |
|  |  | Reverse Primer | CTTTGGAATTGCCTGCACCC |
| HXK2 | NM_001247477.2 | Forward Primer | CAAGCACGCGAATGATGGTT |
|  |  | Reverse Primer | TCTTTGCACCCTTTTCCAGA |
| IPT1 | Solyc05g009410 | Forward Primer | CCTCGCTACTCGTGGTTTCA |
|  |  | Reverse Primer | ATGCCGGAAATCAGAAGGGG |
| IPT2 | Solyc04g007240 | Forward Primer | GGTGAGTGAGGAGCTTCGTT |
|  |  | Reverse Primer | CGGAATCGGAAAACCCCTCT |
| IPT3 | Solyc01g080150 | Forward Primer | ATTGGCTTGCTGTCAACGTC |
|  |  | Reverse Primer | GGAGGGGGAGGAGGGATATT |
| IPT4 | Solyc09g064910 | Forward Primer | TTCCGTGGACAGGCAAACTC |
|  |  | Reverse Primer | TTTCTGCTCTGTCCCATCACC |
| IPT5 | Solyc11g066960 | Forward Primer | GCTCTTGTGAGTCCATTCCT |
|  |  | Reverse Primer | GTTCTGCCCTCTACAGTCTTCTC |
| CYP735A-1 | Solyc02g094860 | Forward Primer | AGCCATCATTTGCATCAAAATCTC |
|  |  | Reverse Primer | ATCGAGGTCTAGGACCACACA |
| CYP735A-2 | Solyc02g094860 | Forward Primer | CCGGTCCTCGGAATTGTGTT |
|  |  | Reverse Primer | CTCCAACTTCCTCCTCAAAAGC |
| CKX5 | Solyc04g016430 | Forward Primer | CGCGAAGGATTTCGGCAAC |
|  |  | Reverse Primer | CTGCATGGTGGAAACGCATC |
| CKX7 | NM_001257979.1 | Forward Primer | CTGCATGGTGGAAACGCATC |
|  |  | Reverse Primer | TCCTCTGTCCTGGAGCAAGT |
| LOG1 | XM_004251100.4 | Forward Primer | CAAGCAGTCTACAGTGGGGG |
|  |  | Reverse Primer | ATTCCGAGTTGAGACCACGC |
| LOG3 | NM_001324502.1 | Forward Primer | TACCAGGTGGCTATGGGACA |
|  |  | Reverse Primer | TGAGTTCTTGGGCAGTTGGG |
| LOG5 | XM_004245030.4 | Forward Primer | TGGCCACAGGAAAGTCTTCA |
|  |  | Reverse Primer | GCCATTTCAGCTTTCCGCTC |

# Supplementary Figure

**Supplementary** **Figure S1.** **The pictures of experimental procedure for CF fluorescence observation. (A)**The cutting end of peduncles about 0.5 cm were soaked in CFDA working solution. **(B)**To protect it from light, the centrifuge tubes were wrapped with tin foil. **(C)** A group of comparison was formed using fruits of similar size. At least three comparison groups were observed.


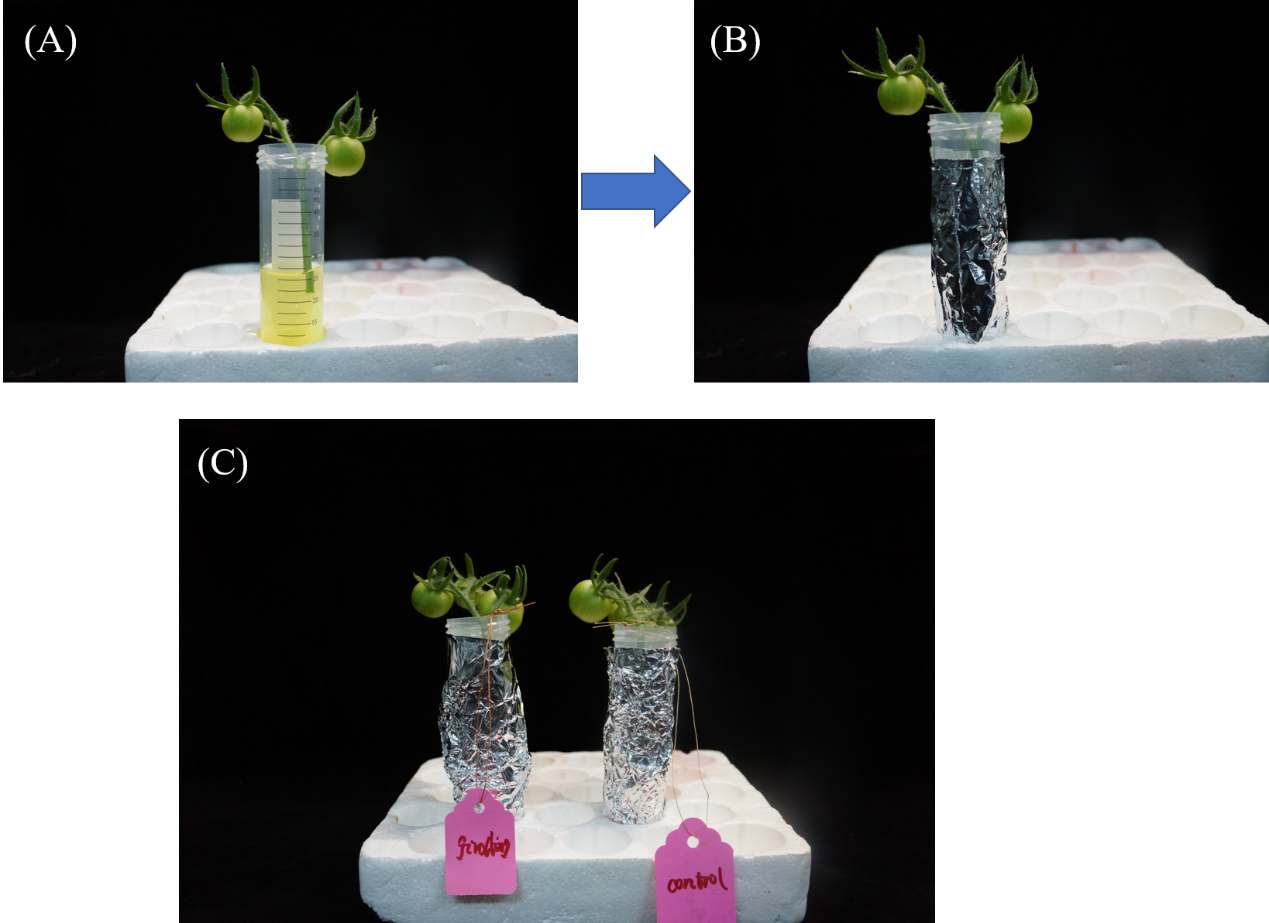


**Supplementary Figure S2.** **Observation of phloem streaming to fruits using CFDA.** CF fluorescence signal was observed at other two groups. In each group, fruit sizes of gridling and control were similar. The fruits in group 3 were larger than those in group 2. Fluorescent image of a free hand cross section of peduncle in control **(A, E)** and in girdling treatment **(B, F)**. Reflected light picture of the same region of control **(C, G)** and of girdling treatment **(D, H)**. The fluorescent image was acquired using a 488 nm excitation laser, the exposure time 3.03 ms and gain number 1.4.


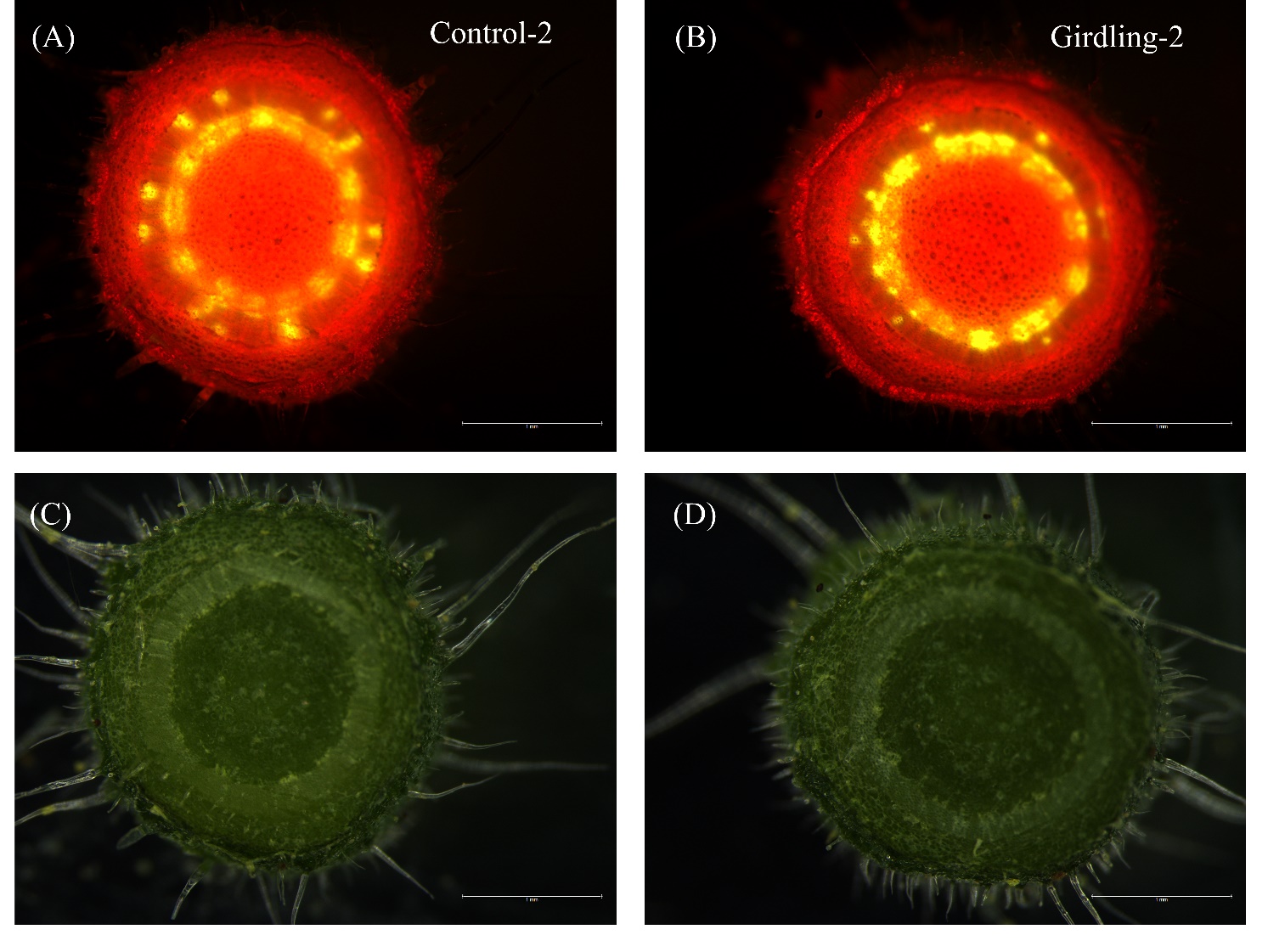


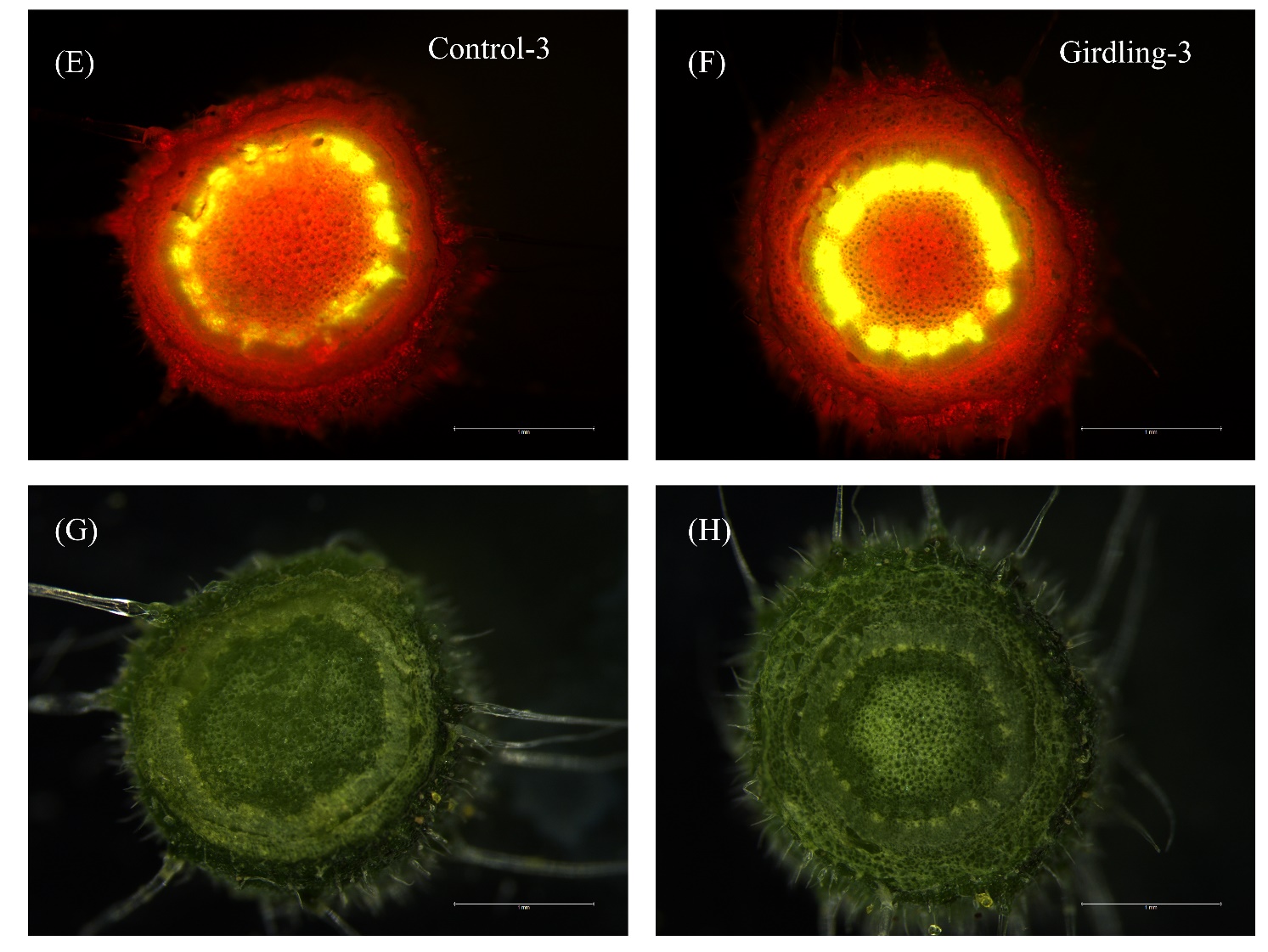


**Supplementary Figure S3. Quantitative analysis of fluorescence intensity.** The CFDA fluorescence signal is quantified using the ImageJ software (ImageJ, RRID:SCR_003070). Asterisks denote significant difference between girdling treatment and control by Student *t* test at p < 0.05, n = 3.





**Supplementary Figure S4. Endogenous level of IAA and GA_3_ in tomato fruit.** The concentration of IAA **(A)** and GA_3_ **(B)** in fruit from 3 to 12 DAT. Asterisks denote significant difference between girdling treatment and control by Student *t* test at p < 0.05, n = 3.
